# Clinical Characteristics and Outcomes of Patients with Constrictive Pericarditis Following Pericardiectomy: An Observational Study

**DOI:** 10.31729/jnma.v63i2091.9236

**Published:** 2025-11-30

**Authors:** Praman Sharma, Lokesh Yadav, Samarpan Timilsina, Subhadra Agrawal, Amritraj Pokhrel, Seema Kumari Chaudhary

**Affiliations:** 1 Department of Cardiothoracic and Vascular Surgery, Nobel Medical College and Teaching Hospital, Biratnagar, Nepal; 2 Department of General Surgery, Nobel Medical College and Teaching Hospital, Biratnagar, Nepal; 3 Department of Obstetrics and Gynecology, Nobel Medical College and Teaching Hospital, Biratnagar, Nepal; 4 Clinical Trial Unit, Oxford University Clinical Research Unit Nepal, Lalitpur, Nepal

**Keywords:** *constrictive pericarditis*, *tuberculosis*, *outcome*

## Abstract

**Introduction:**

Pericardiectomy remains the standard treatment in constrictive pericarditis. The study was aimed to assess the clinical characteristics, etiology and the outcome of patients who underwent pericardiectomy for chronic constrictive pericarditis.

**Methods:**

Single center based retrospective cohort study was conducted on the patients who underwent standard pericardiectomy at our center from January 2021 to December 2023. Structured questionnaire was used to observe the record of the participants. Data was entered in Epi-data and exported to IBM SPSS Statistics version 16 for analysis. Ethical approval was obtained from Institute Review Committee, Nobel Medical College and Teaching Hospital (Ref no: 121/2024).

**Results:**

The study involved 17 patients, with a mean age of 41.76±13.16 years. Male were higher in number with 12 (70.60%) of total cases. The echocardiography findings included annulus reversus, septal bounce, calcified pericardium, and congested Inferior Vena-Cava. Post-operatively, all 17 patients improved to New York Heart Association (NYHA) function class I or II (11 and 6 patients respectively) from (NYHA) functional class III or IV. The average intensive care unit stay was 3±0.7 days and hospital stay were 9.88±2.86 days. Histopathology report revealed tuberculosis in 15 (88.23%) cases.

**Conclusions:**

The most common cause of constrictive pericarditis was tuberculosis and symptomatic improvement was noticed in all patients.

## INTRODUCTION

Constrictive Pericarditis (CP) is a serious condition which leads to chronic pericardial inflammation causing dense fibrosis and calcification of the pericardium.^1^ The most common cause of constrictive pericarditis in Asia and sub-Saharan Africa is tuberculosis.^2^ 50-90% of effusive pericarditis in endemic areas is caused by Tubercular Constrictive Pericarditis (TCP).^3^ Without timely and efficient treatment, chronic pericarditis can progress to life-threatening complication like chronic constrictive pericarditis (CCP), cardiac tamponade, and even death.^4^

Surgical Pericardiectomy is a procedure performed to remove the pericardium, the fibrous sac surrounding the heart in CCP i.e, inflammatory pathology persisting for more than three months in Constrictive Pericarditis (CP), considered the gold standard treatment.^5-8^ Thickened and fibrotic pericardium restricts the heart’s ability to expand fully during diastole, inadequate cardiac filling causing venous congestion which leads to heart failure (HF).^9^ There is a paucity of data on outcomes of CCP in our population, hence this study was conducted.

The study was aimed to assess the surgical outcomes, etiology, post-operative complications, and New York Heart Association (NYHA)^10^ functional class in patients undergoing pericardiectomy for CCP.

## METHODS

Hospital based retrospective study was conducted at Nobel Medical College and Teaching Hospital. The ethical approval for implementation of study was taken from Institutional Review Committee (IRC) of Nobel Medical College and Teaching Hospital (Ref no: 121/2024). This retrospective study included patients diagnosed with constrictive pericarditis with past history of pulmonary tuberculosis who underwent surgical treatment between January 2021 and December 2024 at Nobel Medical College and Teaching Hospital.

Inclusion criteria of this study were confirmed diagnosis of CCP via clinical, echocardiographic, and hemodynamic findings, along with a history or evidence of tuberculosis infection. Patients of age more than or equal to 16 years were considered for enrollment in this study. Patients with CCP with other noncorrective comorbidities were excluded from this study. Patients with incomplete follow-up data or missing clinical records were excluded in this study.

All patients underwent standard pericardiectomy via a median sternotomy approach. The procedure involved careful excision of the thickened and fibrotic pericardium, extending anteriorly from right phrenic nerve to left phrenic nerve and including the diaphragmatic surface of the heart, with the goal of achieving maximal decortication. Cardiopulmonary bypass was not required as all the steps were performed in a beating heart. Intraoperative parameters, including the extent of pericardial resection, duration of surgery, and any operative complications, were meticulously recorded. In cases where dense adhesions were present, mainly over posterior and lateral surfaces of the ventricles, partial pericardiectomy was performed to minimize the risk of injury to underlying myocardial or adjacent vital structures.

The diagnosis of CCP was determined mainly by combination of clinical symptoms, imaging findings (echocardiography and Computed Tomography) and central venous pressure (CVP). CCP was suspected in patients presenting with characteristic clinical signs of right heart failure and was further supported by specific echocardiographic and CT findings. The final confirmation of CCP was achieved intraoperatively during pericardiectomy. The diagnosis of tuberculous pericarditis was based on bacteriological detection, and pathological examination of pericardial tissue.

This study evaluated the clinical symptoms and signs in patients diagnosed with constrictive pericarditis: chest discomfort, peripheral edema, ascites, dyspnea, and fatigability. Symptom severity was assessed using the New York Heart Association (NYHA) functional classification system.^10^ In addition to subjective symptoms, the objective clinical signs: peripheral edema, pericardial knock, pulsus paradoxus, distended neck veins, and Kussmaul’s sign were also documented. Responders to pericardiectomy were defined as patients who demonstrated symptomatic improvement to NYHA Class II or lower at postoperative follow-up. Primary outcomes included survival rates at 3 months. Secondary outcomes encompassed postoperative complications, length of hospital stay, and symptom recurrence.

Data collected included demographic information, clinical presentation, diagnostic imaging, operative details, and postoperative outcomes. Follow-up data were obtained from medical records and patient interviews, focusing on survival rates, symptom relief, and functional status. Structured questionnaire was used for data collection. Pilot survey was performed with 10% of the study participants before conducting the study and the errors in the questionnaire was corrected thereafter.

After data collection, the data was checked for completeness, compiled and entered daily after the field activities in the EPI-DATA software (Version 3.1). The data was then exported to SPSS (Version 16) for further analysis. As the data collected and entered were quantitative, it was analyzed using descriptive statistics as frequencies, percentage, and mean.

## RESULTS

A total of 17 patients with constrictive pericarditis who underwent pericardiectomy were included in the study. All pericardiectomy was performed on beating heart and did not require cardiopulmonary bypass. The mean age of the participants was 41.76±13.16 years with the age ranging from 23 years to 60 years. There were 12 (70.60%) male participants and 5 (29.40%) female participants. Observing the comorbidities, thyroid disorders were found in 5 (29.40%) patients, hypertension in 3 (17.60%) patients, and diabetes in 3 (17.60%) patients. Two (11.80%) participants were smokers.

Preoperatively, 17 (100%) patients demonstrated congested inferior vena-cava (IVC) and calcified pericardium, both of which were absent after the procedure in these patients. Septal bounce which was present in all 17 (100%) patients before surgery was present in 2 (11.76%) participants after surgery (Table 1).

**Table 1:** Clinical and echocardiographic features of patient with constrictive pericarditis (n=17).

Clinical / Echo cardiographic Features	Pre operative n(%)	Post operative n(%)
Congested IVC	17(100%)	0(0%)
Calcified pericardium	17(100%)	0(0%)
Septal bounce	17(100%)	2(11.76%)
Pericardial effusion	2(11.76%)	0(0%)
Annulus reverses	15(88.23%)	3(17.64%)
**NYHA Classification**		
Class I	0(0%)	11(64.70%)
Class II	0(0%)	6(35.29%)
Class III	13(76.47%)	0(0%)
Class IV	4(23.52%)	0(0%)

IVC = Inferior Venacava; NYHA = New York Heart Association

Out of the total patients 11 (64.71%) required ventilator support for less than 24 hours and the mean Intensive Care Unit (ICU) stay was 3.0±0.7 with minimum of 3 and maximum of 5 days stay. The mean hospital stay was 9.88±2.6 with minimum of 7 and maximum of 17 days.

All 17 (100.00%) patients had gone through the tuberculosis test during histopathological examination of pericardial tissues. Among them, 15 (88.23%) participants tested positive for tuberculosis in histopathological examination and remaining 2 (11.76%) patients tested negative.

## DISCUSSION

In low- and middle-income countries, tuberculosis (TB) remains the leading cause of constrictive pericarditis (CP). ^11,12^ Despite receiving adequate anti-tuberculous treatment, only about half of the patients with tuberculous pericardial effusion achieve complete resolution without progressing to constrictive disease.^5,13^ The reasons for this discrepancy remain unclear and may involve host immune response variability, delays in diagnosis, or differing adherence to treatment.

Surgical pericardiectomy is the mainstay of treatment for CCP, especially in cases unresponsive to medical therapy.^3^ Based on the extent of pericardial excision, the procedure is typically classified into partial or total pericardiectomy, with the latter being associated with lower perioperative mortality and improved long-term outcomes.^14^ In our study, all patients underwent total pericardiectomy, aligning with studies recommending complete excision in all cases of CCP not requiring Cardiopulmonary Bypass. ^5,6,11,15^

Patients commonly presented with advanced heart failure (NYHA class III-IV), ascites, peripheral edema, pleural effusion, hepatomegaly, and elevated jugular venous pressure. These symptoms reflect the hemodynamic burden of CP and underscore the importance of early surgical intervention. Short term surgical outcomes were assessed using improvement in NYHA grade and clinical parameters.

The mean patient age was 41.76 years, slightly older than the cohorts reported in Indian studies.^6^ This may suggest a regional variation in the age distribution of TB-related CCP, possibly due to earlier presentation and detection of complication. Similar to prior studies, our cohort showed a male predominance, with a male-to-female ratio exceeding 2:1.^11,14,17^ While past studies have reported ratios ranging from 1.5:1 to 3.4:1, no definitive explanation exists for this gender disparity. Interestingly, we observed lower morbidity among female patients, although this finding warrants further investigation.

The etiology of CP differs by geographic region. In developed countries, idiopathic causes, cardiac surgery, radiation therapy, and prior pericarditis account for the majority of the cases.^17^ In contrast, tuberculosis remains the predominant cause in our setting. In our cohort, 88.23% of patients had CCP confirmed to be of tubercular origin in histopathological examination. This figure is higher than previously reported from other TB-endemic countries such as China (65%), Ghana (63.6%), India (61%), South Korea (42.4%) and Turkey (38%).^12,18^

Echocardiography remains the first-line, non-invasive tool for diagnosing CCP and distinguishing it from restrictive cardiomyopathy.^11,16^ However, CT and MRI provide more detailed anatomical imaging, especially for identifying pericardial calcification and thickening in early disease. In our study, all patients underwent CT, with 17 (100%) showing pericardial thickening or calcification, supporting the role of advanced imaging in early diagnosis.

Tissue Doppler Imaging (TDI), specifically mitral annular velocity measurements, is useful in distinguishing CP from restrictive cardiomyopathy. In CP, the medial mitral annular diastolic velocity (septal e’) is typically preserved or elevated, while the lateral e’ is reduced, a phenomenon known as “annulus reversus”.^19^ In our study, 15 (88.23%) patients showed annulus reversus, a higher percentage than in Indian (60%) and Western cohorts (74%).^19^

Every patient on this study had pericardiectomy on a beating heart without requiring a cardiopulmonary bypass, demonstrating the safety and usefulness of this method, which circumvents the risks associated with extracorporeal movement. The majority of patients (64.70%) were extubated within 24 hours, whereas the remainder required prolonged ventilator support. Comparable trends have been reported in previous studies, where 60-75% of patients achieved early extubation.^21,22^ These findings suggest that early surgical intervention on a beating heart, combined with optimized perioperative management, facilitates favorable recovery, reduces complications, and shortens hospitalization, in line with outcomes documented in the literature.

Despite CCP’s historically high mortality, no deaths were reported in our cohort during the in-hospital period or 90-day follow-up, indicating the potential benefits of early surgical intervention and improved perioperative care.^23^ These results highlight the value of early diagnosis, timely referral for surgery, and standardized perioperative care in enhancing patient outcomes.

This study has several limitations. First, this is a single-center retrospective study in a limited number ofpatients, it is inherently subject to selection bias. Second, the retrospective design limits access to certain key data, such as duration of anti-tuberculosis therapy, incidence of prolonged intubation, and duration of inotropic support. Lastly, long-term outcomes beyond the 90-day post-operative period were not assessed, underscoring the need for future prospective, multi-center studies with extended follow-up to validate these findings.

## CONCLUSION

The most common cause of constrictive was tuberculosis in this study. The patient had NYHA classification of III and VI during presentation which improved after surgery. The majority of patient stayed for less than 24 hours in ICU.

## Data Availability

Data will be made available upon request.
